# Summer Complaint

**Published:** 1856-08

**Authors:** 


### SUMMER COMPLAINT.

How many a sweet child is torn from the arms of its parents, in a great city like this, every day during summer, the "Weekly Reports" fully testify. And what hopes are blasted thereby; what wounds are made, never to be wholly healed on earth; what houses are desolated; what hearts broken; never can be known. But we know, that a large part of all this is avoidable. To those who cannot leave the city, we recommend to keep their children in the second or third stories of their dwellings, until after breakfast, and to confine them to the same from sundown until the next morning, for the general reason, that the most unwholesome atmosphere, mornings and evenings, is at the surface of the earth. The higher we sleep, the healthier.

To those who can go away, we most earnestly say, do not wait an hour, but take your little ones to the sea shore, somewhere. Even if you do not expect them to live a day, not an hour, go along! The change of an hour's distance from New York, in the direction of the sea, is often miraculous.

FAR ROCKAWAY, we greatly prefer to all others in the vicinage of New York, for the following reasons: 1st, It is accessible by four trains every day; only twenty-one miles distant, twelve in the cars, and nine by staging over a plank road, the whole distance being performed in three hours, for seventy-five cents. 2d. Musquito bars are not needed, according to our experience, nor have we seen sand-flies or gnats there. 3d. A very great consideration is, it is out of the way of the rabble. The proprietors of the place having judiciously interdicted the landing of steamboats there, and being reached by stages only, no great numbers can come at any one time. There is not a spot within any reasonable distance of New York, so wholly free from rollicking and rowdy people. And being as it is, on the very shore of the veritable Atlantic, with its beautiful, hard, white beach, where one can promenade or sit and gaze for hours in quiet, we consider it altogether delightful. The accommodations are various, from five to fifteen dollars a week. To those who can afford it, we recommend "*The Pavilion*," which is well kept, and accommodates six hundred persons comfortably. There is preaching in its hall every Sunday. There is a quiet respectability about the whole establishment, which at once commends it to the eye of an observant traveller. An excellent physician visits the hotel regularly every day; and fresh, pure rich milk is abundantly provided twice a day, for the special benefit of sick children.

These remarks are made without fee or reward, past or prospective. We do not even know the names of the proprietors; and we have only written this article in the wish that it may save to other families, as it saved to ours last year, a darling child, whose loss to us would have thrown a cloud over our future existence which no sunshine could have ever dispersed, which no after gladness could ever have driven away.

